# Propane-1,2-diammonium bis­(pyridine-2,6-dicarboxyl­ato-κ^3^ *O*,*N*,*O*′)nickelate(II) tetra­hydrate

**DOI:** 10.1107/S1600536808016309

**Published:** 2008-06-07

**Authors:** Hossein Aghabozorg, Mohammad Heidari, Sara Bagheri, Jafar Attar Gharamaleki, Mohammad Ghadermazi

**Affiliations:** a Faculty of Chemistry, Tarbiat Moallem University, 49 Mofateh Avenue, Tehran, Iran; b Department of Chemistry, Islamic Azad University, North Tehran Branch, Tehran, Iran; c Department of Chemistry, Faculty of Science, University of Kurdistan, Sanandaj, Iran

## Abstract

The reaction of nickel(II) nitrate hexa­hydrate, propane-1,2-diamine and pyridine-2,6-dicarboxylic acid in a 1:2:2 molar ratio in aqueous solution resulted in the formation of the title compound, (C_3_H_12_N_2_)[Ni(C_7_H_3_NO_4_)_2_]·4H_2_O or (*p*-1,2-daH_2_)[Ni(pydc)_2_]·4H_2_O (where *p*-1,2-da is propane-1,2-diamine and pydcH_2_ is pyridine-2,6-dicarboxylic acid). The geometry of the resulting NiN_2_O_4_ coordination can be described as distorted octa­hedral. Considerable C=O⋯π stacking inter­actions are observed between the carboxyl­ate C=O groups and the pyridine rings of the (pydc)^2−^ fragments, with O⋯π distances of 3.1563 (12) and 3.2523 (12) Å and C=O⋯π angles of 95.14 (8) and 94.64 (8)°. In the crystal structure, a wide range of non-covalent inter­actions, consisting of hydrogen bonding [O—H⋯O, N—H⋯O and C—H⋯O, with *D*⋯*A* distances ranging from 2.712 (2) to 3.484 (2) Å], ion pairing, π–π [centroid-to-centroid distance = 3.4825 (8) Å] and C=O⋯π stacking, connect the various components to form a supra­molecular structure.

## Related literature

For related literature, see: Aghabozorg *et al.* (2007 ▶); Aghabozorg, Ghadermazi & Attar Gharamaleki (2006 ▶); Aghabozorg, Ghadermazi & Ramezanipour (2006 ▶); Aghabozorg, Heidari *et al.* (2008 ▶); Aghabozorg, Manteghi & Sheshmani (2008 ▶).
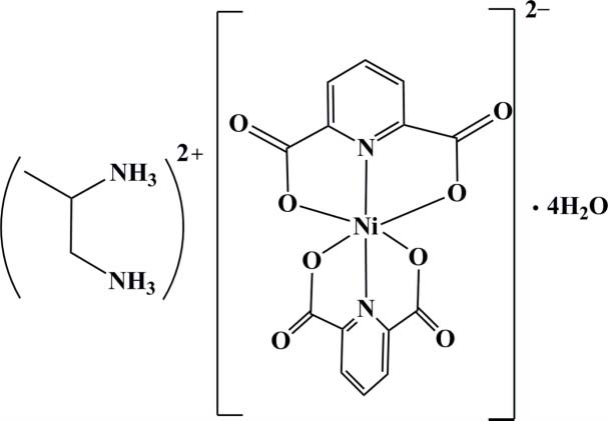


## Experimental

### 

#### Crystal data

(C_3_H_12_N_2_)[Ni(C_7_H_3_NO_4_)_2_]·4H_2_O
*M* *_r_* = 537.13
Orthorhombic, 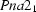

*a* = 20.7598 (6) Å
*b* = 8.2582 (2) Å
*c* = 12.7242 (4) Å
*V* = 2181.42 (11) Å^3^
*Z* = 4
Mo *K*α radiation
μ = 0.96 mm^−1^
*T* = 100 (2) K
0.26 × 0.22 × 0.11 mm

#### Data collection

Bruker APEXII CCD area-detector diffractometer
Absorption correction: multi-scan (*SADABS*; Sheldrick, 1996 ▶) *T*_min_ = 0.781, *T*_max_ = 0.898
36654 measured reflections
6379 independent reflections
6016 reflections with *I* > 2σ(*I*)
*R*_int_ = 0.035

#### Refinement

*R*[*F* ^2^ > 2σ(*F* ^2^)] = 0.023
*wR*(*F* ^2^) = 0.059
*S* = 1.01
6379 reflections
310 parameters
1 restraint
H-atom parameters constrained
Δρ_max_ = 0.34 e Å^−3^
Δρ_min_ = −0.33 e Å^−3^
Absolute structure: Flack (1983 ▶), 2846 Friedel pairs
Flack parameter: 0.004 (7)

### 

Data collection: *APEX2* (Bruker, 2007 ▶); cell refinement: *SAINT* (Bruker, 2007 ▶); data reduction: *SAINT*; program(s) used to solve structure: *SHELXS97* (Sheldrick, 2008 ▶); program(s) used to refine structure: *SHELXL97* (Sheldrick, 2008 ▶); molecular graphics: *SHELXTL* (Sheldrick, 2008 ▶); software used to prepare material for publication: *SHELXTL*.

## Supplementary Material

Crystal structure: contains datablocks I, global. DOI: 10.1107/S1600536808016309/su2054sup1.cif

Structure factors: contains datablocks I. DOI: 10.1107/S1600536808016309/su2054Isup2.hkl

Additional supplementary materials:  crystallographic information; 3D view; checkCIF report

## Figures and Tables

**Table 1:** Hydrogen-bond geometry (Å, °)

*D*—H⋯*A*	*D*—H	H⋯*A*	*D*⋯*A*	*D*—H⋯*A*
O1*W*—H1*WA*⋯O4*W*	0.82	1.97	2.788 (2)	173
O1*W*—H1*WB*⋯O2	0.82	2.47	3.109 (2)	135
O1*W*—H1*WB*⋯O6	0.82	2.21	2.912 (2)	144
O2*W*—H2*WA*⋯O3	0.82	2.21	2.759 (2)	125
N3—H3*B*⋯O4^i^	0.91	1.90	2.795 (2)	168
N3—H3*C*⋯O1*W*	0.91	1.91	2.763 (2)	155
N3—H3*D*⋯O8^ii^	0.91	1.88	2.783 (2)	176
O2*W*—H2*WB*⋯O1^iii^	0.82	2.07	2.849 (2)	160
N4—H4*B*⋯O3*W*^ii^	0.91	1.92	2.812 (2)	165
N4—H4*C*⋯O1	0.91	1.92	2.813 (2)	168
N4—H4*D*⋯O4*W*^iv^	0.91	1.91	2.777 (2)	160
O3*W*—H3*WA*⋯O8	0.82	2.03	2.771 (2)	149
O3*W*—H3*WB*⋯O4^v^	0.82	1.99	2.787 (2)	166
O4*W*—H4*WA*⋯O2*W*^i^	0.82	1.99	2.749 (2)	153
O4*W*—H4*WB*⋯O5	0.82	1.90	2.712 (2)	171
C10—H10*A*⋯O6^vi^	0.95	2.54	3.289 (2)	136
C11—H11*A*⋯O1*W*^vi^	0.95	2.58	3.484 (2)	160
C15—H15*B*⋯O5^vii^	0.99	2.30	3.268 (2)	164
C16—H16*A*⋯O7^ii^	1.00	2.49	3.291 (2)	137

Symmetry codes: (i) 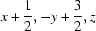; (ii) 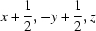; (iii) 
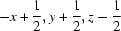
; (iv) 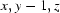; (v) 
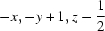
; (vi) 
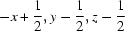
; (vii) 
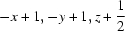
.

**Fig. 1.**
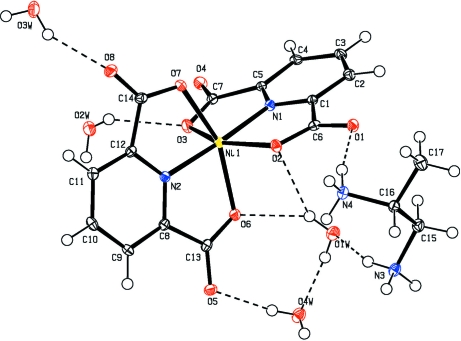
The molecular structure of the title compound with displacement ellipsoids drawn at the 50% probability level. Hydrogen bonds are shown as dashed lines.

**Fig. 2.**
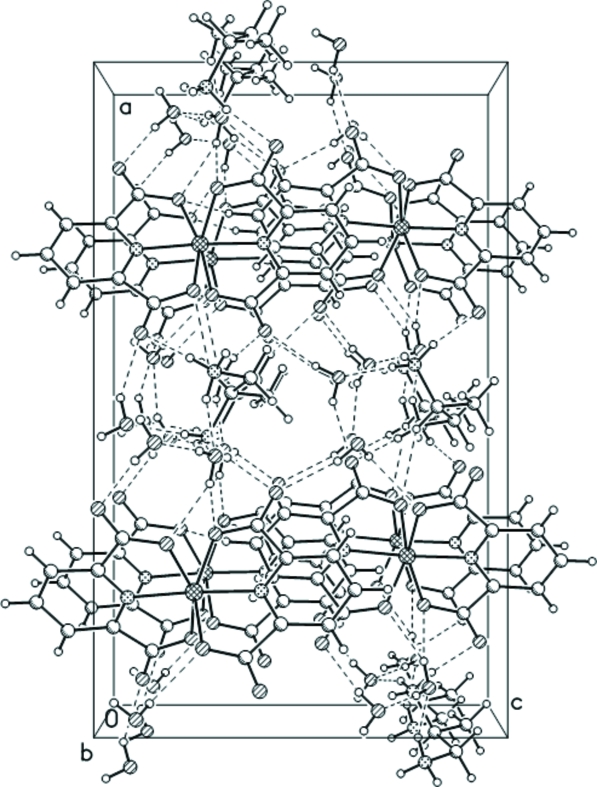
The crystal packing of the title compound as viewed approximately down b, with the hydrogen bonds shown as dashed lines.

**Fig. 3.**
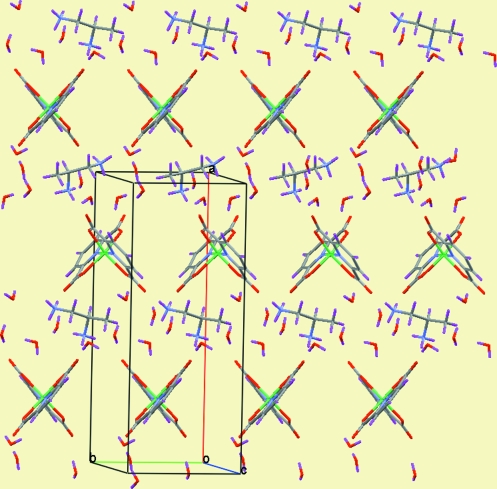
A layered packing diagram of the title compound. The space between the two layers of [Ni(pydc)2]2- fragments is filled with a layer of (p-1,2-daH2)2+ cations and water molecules.

**Fig. 4.**
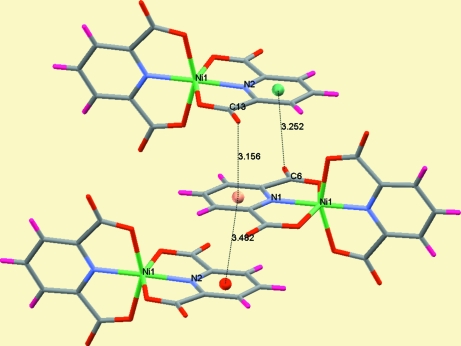
π–π Stacking interaction between two aromatic rings of (pydc)2- units, with centorid–centroid distance of 3.4825 (8) Å (1/2 - x, -1/2 + y, -1/2 + z); C–O···π Stacking interactions between C═O groups of carboxylate and the aromatic rings of pyridine-2,6-dicarboxylate with O···π distances of 3.1563 (12) Å for C13—O5···Cg1 (1/2 - x, 1/2 + y, -1/2 + z) and 3.2523 (12) Å for C6—O1···Cg2 (1/2 - x, -1/2 + y, 1/2 + z) [Cg1 and Cg2 are the centroids for rings N1/C1–C5 and N2/C8–C12, respectively].

## supplementary crystallographic information

### Comment

Recently, we have defined a plan to prepare water soluble proton transfer compounds as novel self-assembled systems that can function as suitable ligands in the synthesis of metal complexes. In this regard, we have reported cases in which proton transfers from pyridine-2,6-dicarboxylic acid (pydcH_2_), and benzene-1,2,4,5-tetracarboxylic acid (btcH_4_), to propane-1,3-diamine (pda), propane-1,2-diamine (*p*-1,2-da) and 1,10-phenanthroline, (phen). This work has resulted in the formation of some novel proton transfer compounds such as (pdaH_2_)(pydc).(pydcH_2_).2.5H_2_O (Aghabozorg, Ghadermazi & Ramezanipour, 2006), (pdaH_2_)_2_(btc).2H_2_O (Aghabozorg *et al.*, 2007), (*p*-1,2-daH_2_)(pydcH)_2_.2H_2_O (Aghabozorg, Heidari *et al.*, 2008) and (phenH)_4_(btcH_3_)_2_(btcH_2_) (Aghabozorg, Ghadermazi & Attar Gharamaleki, 2006). For more details and related literature see our recent review article (Aghabozorg, Manteghi & Sheshmani, 2008).

The molecular structure and crystal packing diagram of the title compound are presented in Figs. 1 and 2, respectively. The Ni^II^ atom is six-coordinated by two pyridine-2,6-dicarboxylate, or (pydc)^2-^, groups, *i.e.* each (pydc)^2-^ ligand is coordinated through one pyridine N atom and two carboxylate O atoms. As it can be seen, atoms N1 and N2 of the two (pydc)^2-^ fragments occupy the axial positions, while atoms O2, O3, O6 and O7 form the equatorial plane [with Ni—O distances ranging from 2.1178 (11) to 2.1477 (10) Å]. The N1—Ni1—N2 angle [176.17 (5)°] deviates from linearity. Therefore, the geometry of the resulting NiN_2_O_4_ coordination can be described as distorted octahedral. The O2—Ni1—O6 and O3—Ni1—O7 bond angles are equal to 87.26 (4)° and 90.55 (4)°, respectively. On the other hand, the torsion angles O3—Ni1—O7—C14 and O7—Ni1—O3—C7 are 92.68 (10)° and 95.05 (10)°, respectively, indicating that the two (pydc)^2-^ units are almost perpendicular to one another. The O2—Ni1—O3 [155.41 (4)°] and O6—Ni1—O7 [155.96 (4)°] bond angles indicate that the four carboxylate groups of the two dianions are oriented in a flattened tetrahedral arrangement around the Ni1 atom.

It is interesting to note that the crystal packing shows a layered structure. The space between the layers of [Ni(pydc)_2_]^2-^ units is occupied by (*p*-1,2-daH_2_)^2+^ cations and uncoordinated water molecules, which bridge the [Ni(pydc)_2_]^2-^ units *via* hydrogen bonds (Fig 3 and Table 1). A noticeable feature of the title compound is the presence of C═O···π stacking interactions, between C═O groups of the carboxylate with aromatic rings of pyridine-2,6-dicarboxylate, with O···π distances of 3.1563 (12) Å for C13–O5···*Cg*1 (1/2 - *x*, 1/2 + *y*, -1/2 + *z*) and 3.2523 (12) Å for C6–O1···*Cg*2 (1/2 - *x*, -1/2 + *y*, 1/2 + *z*) [*Cg*1 and *Cg*2 are the centroids of the rings N1/C1–C5 and N2/C8–C12, respectively]. There is also considerable π–π stacking interactions between the two aromatic rings of the (pydc)^2-^ units, with a centorid–centroid distance of 3.4825 (8) Å (1/2 - *x*, -1/2 + *y*, -1/2 + *z*) [see Fig. 4]. In the crystal structure, a wide range of non-covalent interactions consisting of hydrogen bonding (of the type O—H···O, N—H···O and C—H···O with D···A ranging from 2.712 (2) Å to 3.484 (2) Å), ion pairing, π···π and C═ O···π stacking connect the various components to form a supramolecular structure.

### Experimental

An aqueous solution of Ni(NO_3_)_2_.6H_2_O (290 mg, 1 mmol), propane-1,2-diamine (80 mg, 2 mmol) and pyridine-2,6-dicarboxylic acid (360 mg, 2 mmol) was added to each other in a 1:2:2 molar ratio, and the reaction mixture was heated at about 40°C for 5 h. Green crystals of the title compound were obtained from the solution after four weeks at room temperature.

### Refinement

The hydrogen atoms of the NH3 groups and the water molecules were located in difference Fourier maps. The H(C) atom positions were included in calculated positions and treated as riding atoms with *U*_iso_(H) = 1.2*U*_eq_(parent C or O atoms) and 1.5*U*_eq_(parent N or C-methyl atoms).

### Crystal data

**Table:**

(C_3_H_12_N_2_)[Ni(C_7_H_3_NO_4_)_2_]·4H_2_O	*F*_000_ = 1120
*M**_r_* = 537.13	*D*_x_ = 1.635 Mg m^−^^3^
Orthorhombic, *P**n**a*2_1_	Mo *K*α radiation λ = 0.71073 Å
Hall symbol: P 2c -2n	Cell parameters from 410 reflections
*a* = 20.7598 (6) Å	θ = 3–29º
*b* = 8.2582 (2) Å	µ = 0.96 mm^−^^1^
*c* = 12.7242 (4) Å	*T* = 100 (2) K
*V* = 2181.42 (11) Å^3^	Prism, light-green
*Z* = 4	0.26 × 0.22 × 0.11 mm

### Data collection

**Table:**

Bruker APEXII CCD area-detector diffractometer	6379 independent reflections
Radiation source: fine-focus sealed tube	6016 reflections with *I* > 2σ(*I*)
Monochromator: graphite	*R*_int_ = 0.035
*T* = 100(2) K	θ_max_ = 30.0º
ω scans	θ_min_ = 2.0º
Absorption correction: multi-scan(SADABS; Sheldrick, 1996)	*h* = −29→29
*T*_min_ = 0.781, *T*_max_ = 0.898	*k* = −11→11
36654 measured reflections	*l* = −17→17

### Refinement

**Table:**

Refinement on *F*^2^	Hydrogen site location: mixed
Least-squares matrix: full	H-atom parameters constrained
*R*[*F*^2^ > 2σ(*F*^2^)] = 0.023	*w* = 1/[σ^2^(*F*_o_^2^) + (0.03*P*)^2^ + 0.5*P*] where *P* = (*F*_o_^2^ + 2*F*_c_^2^)/3
*wR*(*F*^2^) = 0.059	(Δ/σ)_max_ = 0.001
*S* = 1.01	Δρ_max_ = 0.34 e Å^−^^3^
6379 reflections	Δρ_min_ = −0.33 e Å^−^^3^
310 parameters	Extinction correction: none
1 restraint	Absolute structure: Flack (1983), with how many Friedel pairs?
Primary atom site location: structure-invariant direct methods	Flack parameter: 0.004 (7)
Secondary atom site location: difference Fourier map	

### Special details

**Table:**

Geometry. All e.s.d.'s (except the e.s.d. in the dihedral angle between two l.s. planes) are estimated using the full covariance matrix. The cell e.s.d.'s are taken into account individually in the estimation of e.s.d.'s in distances, angles and torsion angles; correlations between e.s.d.'s in cell parameters are only used when they are defined by crystal symmetry. An approximate (isotropic) treatment of cell e.s.d.'s is used for estimating e.s.d.'s involving l.s. planes.
Refinement. Refinement of *F*^2^ against ALL reflections. The weighted *R*-factor *wR* and goodness of fit *S* are based on *F*^2^, conventional *R*-factors *R* are based on *F*, with *F* set to zero for negative *F*^2^. The threshold expression of *F*^2^ > σ(*F*^2^) is used only for calculating *R*-factors(gt) *etc*. and is not relevant to the choice of reflections for refinement. *R*-factors based on *F*^2^ are statistically about twice as large as those based on *F*, and *R*- factors based on ALL data will be even larger.

### Fractional atomic coordinates and isotropic or equivalent isotropic displacement parameters (Å^2^)

**Table:**

	*x*	*y*	*z*	*U*_iso_*/*U*_eq_	
Ni1	0.230880 (7)	0.50781 (2)	0.25665 (2)	0.00841 (4)	
O1	0.36657 (5)	0.27840 (12)	0.44207 (8)	0.0123 (2)	
O2	0.30848 (5)	0.35043 (12)	0.30169 (8)	0.0110 (2)	
O3	0.15872 (5)	0.68763 (13)	0.28120 (8)	0.0131 (2)	
O4	0.10593 (5)	0.81657 (13)	0.41025 (9)	0.0133 (2)	
O5	0.35128 (5)	0.74395 (13)	0.04995 (8)	0.0128 (2)	
O6	0.30178 (5)	0.66991 (13)	0.19960 (8)	0.0113 (2)	
O7	0.15889 (5)	0.32530 (12)	0.24551 (9)	0.0120 (2)	
O8	0.09570 (5)	0.20477 (13)	0.12645 (9)	0.0146 (2)	
N1	0.23302 (5)	0.53498 (17)	0.41041 (11)	0.0085 (2)	
N2	0.22246 (6)	0.48264 (15)	0.10356 (12)	0.0090 (3)	
C1	0.27701 (7)	0.45504 (17)	0.46722 (12)	0.0087 (2)	
C2	0.28041 (8)	0.47344 (19)	0.57519 (13)	0.0109 (3)	
H2A	0.3120	0.4178	0.6153	0.013*	
C3	0.23594 (7)	0.57630 (18)	0.62343 (12)	0.0112 (3)	
H3A	0.2371	0.5909	0.6975	0.013*	
C4	0.18997 (7)	0.65754 (17)	0.56366 (11)	0.0102 (3)	
H4A	0.1593	0.7270	0.5958	0.012*	
C5	0.19040 (7)	0.63384 (17)	0.45558 (11)	0.0088 (2)	
C6	0.32098 (6)	0.35197 (16)	0.39952 (12)	0.0096 (2)	
C7	0.14761 (7)	0.71970 (17)	0.37710 (11)	0.0102 (3)	
C8	0.26305 (6)	0.56135 (18)	0.04015 (12)	0.0095 (3)	
C9	0.26076 (7)	0.5380 (2)	−0.06858 (12)	0.0105 (3)	
H9A	0.2901	0.5919	−0.1140	0.013*	
C10	0.21417 (7)	0.43328 (18)	−0.10826 (11)	0.0121 (3)	
H10A	0.2116	0.4147	−0.1818	0.015*	
C11	0.17109 (7)	0.35526 (17)	−0.04071 (12)	0.0110 (3)	
H11A	0.1385	0.2857	−0.0673	0.013*	
C12	0.17748 (6)	0.38260 (17)	0.06599 (12)	0.0097 (2)	
C13	0.30969 (6)	0.66803 (17)	0.10021 (12)	0.0101 (3)	
C14	0.14033 (7)	0.29812 (17)	0.15243 (12)	0.0108 (3)	
N3	0.54006 (6)	0.43977 (15)	0.30200 (10)	0.0123 (2)	
H3B	0.5651	0.5201	0.3289	0.018*	
H3C	0.5010	0.4809	0.2837	0.018*	
H3D	0.5595	0.3971	0.2442	0.018*	
N4	0.44676 (5)	0.16524 (15)	0.27970 (10)	0.0118 (2)	
H4B	0.4502	0.2393	0.2270	0.018*	
H4C	0.4162	0.1982	0.3263	0.018*	
H4D	0.4354	0.0676	0.2523	0.018*	
C15	0.53120 (7)	0.31041 (17)	0.38271 (12)	0.0130 (3)	
H15A	0.4983	0.3459	0.4340	0.016*	
H15B	0.5722	0.2949	0.4212	0.016*	
C16	0.51052 (7)	0.14977 (17)	0.33504 (12)	0.0113 (2)	
H16A	0.5437	0.1154	0.2827	0.014*	
C17	0.50526 (8)	0.01962 (18)	0.41937 (13)	0.0174 (3)	
H17A	0.4940	−0.0840	0.3866	0.026*	
H17B	0.4718	0.0499	0.4700	0.026*	
H17C	0.5466	0.0091	0.4558	0.026*	
O1W	0.42220 (5)	0.59529 (14)	0.30653 (9)	0.0169 (2)	
H1WA	0.4295	0.6786	0.2735	0.020*	
H1WB	0.3837	0.5823	0.2945	0.020*	
O2W	0.06395 (6)	0.74698 (19)	0.13415 (10)	0.0297 (3)	
H2WA	0.0821	0.6693	0.1612	0.036*	
H2WB	0.0882	0.7755	0.0867	0.036*	
O3W	−0.03481 (5)	0.15335 (15)	0.09506 (9)	0.0199 (2)	
H3WA	0.0027	0.1743	0.0800	0.024*	
H3WB	−0.0520	0.1482	0.0372	0.024*	
O4W	0.44262 (5)	0.86620 (13)	0.18005 (9)	0.0158 (2)	
H4WA	0.4731	0.8280	0.1474	0.019*	
H4WB	0.4180	0.8294	0.1357	0.019*	

### Atomic displacement parameters (Å^2^)

**Table:**

	*U*^11^	*U*^22^	*U*^33^	*U*^12^	*U*^13^	*U*^23^
Ni1	0.00786 (7)	0.01104 (7)	0.00634 (7)	0.00044 (6)	−0.00030 (7)	−0.00050 (7)
O1	0.0097 (4)	0.0146 (5)	0.0125 (5)	0.0032 (4)	−0.0006 (4)	0.0011 (4)
O2	0.0123 (4)	0.0127 (5)	0.0082 (5)	0.0015 (4)	−0.0003 (4)	−0.0013 (4)
O3	0.0121 (5)	0.0174 (5)	0.0097 (5)	0.0037 (4)	−0.0006 (4)	0.0003 (4)
O4	0.0125 (5)	0.0146 (5)	0.0129 (5)	0.0047 (4)	0.0020 (4)	0.0016 (4)
O5	0.0110 (5)	0.0154 (5)	0.0121 (5)	−0.0025 (4)	0.0019 (4)	−0.0005 (4)
O6	0.0106 (5)	0.0139 (5)	0.0092 (5)	−0.0009 (4)	−0.0003 (4)	−0.0009 (4)
O7	0.0115 (4)	0.0165 (4)	0.0081 (5)	−0.0015 (3)	0.0002 (4)	0.0010 (4)
O8	0.0131 (5)	0.0173 (5)	0.0134 (5)	−0.0049 (4)	−0.0003 (4)	−0.0013 (4)
N1	0.0095 (6)	0.0079 (5)	0.0080 (6)	−0.0005 (4)	−0.0004 (4)	−0.0014 (5)
N2	0.0081 (5)	0.0099 (5)	0.0088 (6)	0.0014 (4)	0.0000 (5)	0.0009 (5)
C1	0.0084 (6)	0.0083 (6)	0.0095 (6)	−0.0008 (5)	−0.0002 (5)	0.0010 (5)
C2	0.0123 (6)	0.0093 (6)	0.0111 (7)	−0.0004 (5)	−0.0004 (5)	0.0009 (5)
C3	0.0136 (6)	0.0122 (6)	0.0080 (6)	−0.0017 (5)	0.0010 (5)	−0.0003 (5)
C4	0.0106 (6)	0.0110 (6)	0.0090 (6)	−0.0010 (5)	0.0032 (5)	−0.0012 (5)
C5	0.0074 (6)	0.0098 (6)	0.0094 (6)	−0.0007 (5)	0.0001 (5)	0.0019 (5)
C6	0.0084 (6)	0.0084 (6)	0.0121 (6)	−0.0010 (4)	0.0012 (5)	−0.0001 (5)
C7	0.0090 (6)	0.0102 (6)	0.0114 (6)	−0.0010 (5)	−0.0005 (5)	0.0012 (5)
C8	0.0080 (6)	0.0096 (6)	0.0108 (7)	0.0008 (5)	0.0000 (5)	−0.0010 (5)
C9	0.0135 (6)	0.0098 (6)	0.0081 (7)	0.0006 (5)	0.0003 (5)	0.0022 (5)
C10	0.0171 (7)	0.0122 (6)	0.0071 (6)	0.0015 (5)	−0.0011 (5)	−0.0013 (5)
C11	0.0112 (6)	0.0105 (6)	0.0112 (6)	0.0007 (5)	−0.0015 (5)	−0.0004 (5)
C12	0.0073 (6)	0.0098 (6)	0.0121 (6)	0.0011 (5)	0.0001 (5)	−0.0004 (5)
C13	0.0083 (6)	0.0096 (6)	0.0125 (6)	0.0015 (5)	−0.0007 (5)	−0.0010 (5)
C14	0.0088 (6)	0.0116 (6)	0.0119 (6)	0.0010 (5)	0.0022 (5)	0.0001 (5)
N3	0.0108 (5)	0.0121 (5)	0.0141 (6)	−0.0006 (4)	−0.0012 (4)	−0.0033 (5)
N4	0.0106 (5)	0.0127 (5)	0.0122 (6)	0.0001 (4)	−0.0016 (4)	−0.0010 (4)
C15	0.0127 (6)	0.0143 (6)	0.0119 (6)	−0.0011 (5)	−0.0011 (5)	−0.0020 (5)
C16	0.0099 (6)	0.0127 (6)	0.0113 (6)	0.0003 (5)	−0.0007 (5)	−0.0006 (5)
C17	0.0204 (7)	0.0153 (7)	0.0164 (7)	0.0027 (5)	−0.0024 (6)	0.0026 (6)
O1W	0.0118 (5)	0.0196 (5)	0.0193 (6)	0.0011 (4)	−0.0009 (4)	0.0034 (4)
O2W	0.0140 (5)	0.0595 (9)	0.0155 (6)	−0.0035 (6)	−0.0019 (4)	0.0121 (6)
O3W	0.0142 (5)	0.0318 (6)	0.0138 (5)	0.0009 (5)	−0.0037 (4)	−0.0011 (5)
O4W	0.0139 (5)	0.0171 (5)	0.0163 (5)	−0.0014 (4)	−0.0006 (4)	−0.0047 (4)

### Geometric parameters (Å, °)

**Table:**

Ni1—N2	1.9668 (15)	C9—H9A	0.9500
Ni1—N1	1.9698 (14)	C10—C11	1.398 (2)
Ni1—O6	2.1178 (11)	C10—H10A	0.9500
Ni1—O7	2.1273 (10)	C11—C12	1.383 (2)
Ni1—O3	2.1324 (10)	C11—H11A	0.9500
Ni1—O2	2.1477 (10)	C12—C14	1.514 (2)
O1—C6	1.2483 (17)	N3—C15	1.4932 (19)
O2—C6	1.2717 (18)	N3—H3B	0.9100
O3—C7	1.2697 (18)	N3—H3C	0.9100
O4—C7	1.2516 (17)	N3—H3D	0.9100
O5—C13	1.2441 (18)	N4—C16	1.5048 (17)
O6—C13	1.2754 (19)	N4—H4B	0.9100
O7—C14	1.2655 (19)	N4—H4C	0.9100
O8—C14	1.2498 (18)	N4—H4D	0.9100
N1—C5	1.3340 (19)	C15—C16	1.5205 (19)
N1—C1	1.3387 (19)	C15—H15A	0.9900
N2—C12	1.335 (2)	C15—H15B	0.9900
N2—C8	1.335 (2)	C16—C17	1.523 (2)
C1—C2	1.384 (2)	C16—H16A	1.0000
C1—C6	1.516 (2)	C17—H17A	0.9800
C2—C3	1.397 (2)	C17—H17B	0.9800
C2—H2A	0.9500	C17—H17C	0.9800
C3—C4	1.393 (2)	O1W—H1WA	0.8201
C3—H3A	0.9500	O1W—H1WB	0.8200
C4—C5	1.389 (2)	O2W—H2WA	0.8201
C4—H4A	0.9500	O2W—H2WB	0.8198
C5—C7	1.5130 (19)	O3W—H3WA	0.8200
C8—C9	1.398 (2)	O3W—H3WB	0.8201
C8—C13	1.516 (2)	O4W—H4WA	0.8201
C9—C10	1.392 (2)	O4W—H4WB	0.8201
			
N2—Ni1—N1	176.17 (5)	C8—C9—H9A	121.0
N2—Ni1—O6	77.83 (5)	C9—C10—C11	120.52 (14)
N1—Ni1—O6	104.64 (5)	C9—C10—H10A	119.7
N2—Ni1—O7	78.27 (5)	C11—C10—H10A	119.7
N1—Ni1—O7	99.37 (5)	C12—C11—C10	117.85 (14)
O6—Ni1—O7	155.96 (4)	C12—C11—H11A	121.1
N2—Ni1—O3	98.99 (5)	C10—C11—H11A	121.1
N1—Ni1—O3	77.94 (5)	N2—C12—C11	121.31 (14)
O6—Ni1—O3	95.64 (4)	N2—C12—C14	112.42 (14)
O7—Ni1—O3	90.55 (4)	C11—C12—C14	126.11 (13)
N2—Ni1—O2	105.48 (5)	O5—C13—O6	126.37 (14)
N1—Ni1—O2	77.70 (5)	O5—C13—C8	118.49 (13)
O6—Ni1—O2	87.29 (4)	O6—C13—C8	115.14 (12)
O7—Ni1—O2	96.66 (4)	O8—C14—O7	125.63 (14)
O3—Ni1—O2	155.41 (4)	O8—C14—C12	118.02 (13)
C6—O2—Ni1	114.09 (9)	O7—C14—C12	116.31 (12)
C7—O3—Ni1	114.43 (9)	C15—N3—H3B	109.5
C13—O6—Ni1	114.94 (9)	C15—N3—H3C	109.5
C14—O7—Ni1	113.70 (9)	H3B—N3—H3C	109.5
C5—N1—C1	121.44 (14)	C15—N3—H3D	109.5
C5—N1—Ni1	118.86 (10)	H3B—N3—H3D	109.5
C1—N1—Ni1	119.70 (10)	H3C—N3—H3D	109.5
C12—N2—C8	121.75 (15)	C16—N4—H4B	109.5
C12—N2—Ni1	118.82 (11)	C16—N4—H4C	109.5
C8—N2—Ni1	119.39 (11)	H4B—N4—H4C	109.5
N1—C1—C2	121.11 (14)	C16—N4—H4D	109.5
N1—C1—C6	112.38 (13)	H4B—N4—H4D	109.5
C2—C1—C6	126.50 (14)	H4C—N4—H4D	109.5
C1—C2—C3	117.99 (14)	N3—C15—C16	112.62 (12)
C1—C2—H2A	121.0	N3—C15—H15A	109.1
C3—C2—H2A	121.0	C16—C15—H15A	109.1
C4—C3—C2	120.40 (14)	N3—C15—H15B	109.1
C4—C3—H3A	119.8	C16—C15—H15B	109.1
C2—C3—H3A	119.8	H15A—C15—H15B	107.8
C5—C4—C3	117.93 (13)	N4—C16—C15	111.16 (11)
C5—C4—H4A	121.0	N4—C16—C17	109.06 (12)
C3—C4—H4A	121.0	C15—C16—C17	110.80 (12)
N1—C5—C4	121.13 (14)	N4—C16—H16A	108.6
N1—C5—C7	113.07 (13)	C15—C16—H16A	108.6
C4—C5—C7	125.70 (13)	C17—C16—H16A	108.6
O1—C6—O2	125.06 (13)	C16—C17—H17A	109.5
O1—C6—C1	118.90 (13)	C16—C17—H17B	109.5
O2—C6—C1	116.03 (12)	H17A—C17—H17B	109.5
O4—C7—O3	125.65 (13)	C16—C17—H17C	109.5
O4—C7—C5	118.88 (13)	H17A—C17—H17C	109.5
O3—C7—C5	115.46 (12)	H17B—C17—H17C	109.5
N2—C8—C9	120.62 (14)	H1WA—O1W—H1WB	101.2
N2—C8—C13	112.42 (13)	H2WA—O2W—H2WB	104.5
C9—C8—C13	126.95 (13)	H3WA—O3W—H3WB	102.4
C10—C9—C8	117.93 (14)	H4WA—O4W—H4WB	89.5
C10—C9—H9A	121.0		
			
N2—Ni1—O2—C6	179.68 (10)	Ni1—N1—C5—C4	−179.84 (10)
N1—Ni1—O2—C6	−2.52 (10)	C1—N1—C5—C7	176.61 (12)
O6—Ni1—O2—C6	103.10 (10)	Ni1—N1—C5—C7	−3.14 (16)
O7—Ni1—O2—C6	−100.70 (10)	C3—C4—C5—N1	0.7 (2)
O3—Ni1—O2—C6	5.46 (16)	C3—C4—C5—C7	−175.52 (13)
N2—Ni1—O3—C7	173.26 (10)	Ni1—O2—C6—O1	−175.13 (11)
N1—Ni1—O3—C7	−4.41 (10)	Ni1—O2—C6—C1	3.57 (15)
O6—Ni1—O3—C7	−108.21 (10)	N1—C1—C6—O1	175.89 (13)
O7—Ni1—O3—C7	95.05 (10)	C2—C1—C6—O1	−2.4 (2)
O2—Ni1—O3—C7	−12.39 (16)	N1—C1—C6—O2	−2.89 (18)
N2—Ni1—O6—C13	−4.98 (10)	C2—C1—C6—O2	178.85 (14)
N1—Ni1—O6—C13	178.04 (10)	Ni1—O3—C7—O4	−177.39 (11)
O7—Ni1—O6—C13	1.20 (16)	Ni1—O3—C7—C5	4.04 (15)
O3—Ni1—O6—C13	−102.99 (10)	N1—C5—C7—O4	−179.55 (13)
O2—Ni1—O6—C13	101.50 (10)	C4—C5—C7—O4	−3.0 (2)
N2—Ni1—O7—C14	−6.39 (10)	N1—C5—C7—O3	−0.87 (18)
N1—Ni1—O7—C14	170.54 (10)	C4—C5—C7—O3	175.64 (13)
O6—Ni1—O7—C14	−12.55 (15)	C12—N2—C8—C9	−1.7 (2)
O3—Ni1—O7—C14	92.68 (10)	Ni1—N2—C8—C9	176.03 (11)
O2—Ni1—O7—C14	−110.88 (10)	C12—N2—C8—C13	179.89 (12)
O6—Ni1—N1—C5	96.77 (11)	Ni1—N2—C8—C13	−2.41 (16)
O7—Ni1—N1—C5	−84.54 (11)	N2—C8—C9—C10	1.3 (2)
O3—Ni1—N1—C5	4.01 (11)	C13—C8—C9—C10	179.52 (14)
O2—Ni1—N1—C5	−179.38 (12)	C8—C9—C10—C11	0.3 (2)
O6—Ni1—N1—C1	−83.00 (12)	C9—C10—C11—C12	−1.6 (2)
O7—Ni1—N1—C1	95.70 (11)	C8—N2—C12—C11	0.3 (2)
O3—Ni1—N1—C1	−175.75 (12)	Ni1—N2—C12—C11	−177.38 (10)
O2—Ni1—N1—C1	0.86 (11)	C8—N2—C12—C14	175.92 (12)
O6—Ni1—N2—C12	−178.35 (11)	Ni1—N2—C12—C14	−1.80 (16)
O7—Ni1—N2—C12	4.21 (10)	C10—C11—C12—N2	1.3 (2)
O3—Ni1—N2—C12	−84.47 (11)	C10—C11—C12—C14	−173.67 (13)
O2—Ni1—N2—C12	97.96 (11)	Ni1—O6—C13—O5	−174.76 (11)
O6—Ni1—N2—C8	3.88 (10)	Ni1—O6—C13—C8	5.13 (15)
O7—Ni1—N2—C8	−173.56 (11)	N2—C8—C13—O5	177.84 (12)
O3—Ni1—N2—C8	97.75 (11)	C9—C8—C13—O5	−0.5 (2)
O2—Ni1—N2—C8	−79.81 (11)	N2—C8—C13—O6	−2.06 (18)
C5—N1—C1—C2	−0.8 (2)	C9—C8—C13—O6	179.62 (14)
Ni1—N1—C1—C2	179.00 (11)	Ni1—O7—C14—O8	−174.94 (12)
C5—N1—C1—C6	−179.12 (12)	Ni1—O7—C14—C12	7.30 (15)
Ni1—N1—C1—C6	0.63 (16)	N2—C12—C14—O8	178.05 (12)
N1—C1—C2—C3	0.9 (2)	C11—C12—C14—O8	−6.6 (2)
C6—C1—C2—C3	179.00 (13)	N2—C12—C14—O7	−4.02 (18)
C1—C2—C3—C4	−0.2 (2)	C11—C12—C14—O7	171.32 (14)
C2—C3—C4—C5	−0.6 (2)	N3—C15—C16—N4	−61.32 (15)
C1—N1—C5—C4	−0.1 (2)	N3—C15—C16—C17	177.24 (12)

### Hydrogen-bond geometry (Å, °)

**Table:**

*D*—H···*A*	*D*—H	H···*A*	*D*···*A*	*D*—H···*A*
O1W—H1WA···O4W	0.82	1.97	2.788 (2)	173
O1W—H1WB···O2	0.82	2.47	3.109 (2)	135
O1W—H1WB···O6	0.82	2.21	2.912 (2)	144
O2W—H2WA···O3	0.82	2.21	2.759 (2)	125
N3—H3B···O4^i^	0.91	1.90	2.795 (2)	168
N3—H3C···O1W	0.91	1.91	2.763 (2)	155
N3—H3D···O8^ii^	0.91	1.88	2.783 (2)	176
O2W—H2WB···O1^iii^	0.82	2.07	2.849 (2)	160
N4—H4B···O3W^ii^	0.91	1.92	2.812 (2)	165
N4—H4C···O1	0.91	1.92	2.813 (2)	168
N4—H4D···O4W^iv^	0.91	1.91	2.777 (2)	160
O3W—H3WA···O8	0.82	2.03	2.771 (2)	149
O3W—H3WB···O4^v^	0.82	1.99	2.787 (2)	166
O4W—H4WA···O2W^i^	0.82	1.99	2.749 (2)	153
O4W—H4WB···O5	0.82	1.90	2.712 (2)	171
C10—H10A···O6^vi^	0.95	2.54	3.289 (2)	136
C11—H11A···O1W^vi^	0.95	2.58	3.484 (2)	160
C15—H15B···O5^vii^	0.99	2.30	3.268 (2)	164
C16—H16A···O7^ii^	1.00	2.49	3.291 (2)	137

Symmetry codes:  (i) *x*+1/2, −*y*+3/2, *z*; (ii) *x*+1/2, −*y*+1/2, *z*; (iii) −*x*+1/2, *y*+1/2, *z*−1/2; (iv) *x*, *y*−1, *z*; (v) −*x*, −*y*+1, *z*−1/2; (vi) −*x*+1/2, *y*−1/2, *z*−1/2; (vii) −*x*+1, −*y*+1, *z*+1/2.
